# Brain and behavioural responses to food viewing in women during pregnancy and their relationship with metabolic health: study protocol for the FOODY Play Study – a prospective observational study

**DOI:** 10.1136/bmjopen-2026-119379

**Published:** 2026-09-11

**Authors:** Mariana Treviño Montemayor, Amel Hamam-Mechkour, Jennifer J. Eisler, Micah M. Murray, Lucas Spierer, Sybille Schenk, Ryan J. Halter, Ulrike Toepel, Antje Horsch, Chrysa Retsa, Amar Arhab, Dan Yedu Quansah, Jardena J. Puder

**Affiliations:** 1 Obstetric Service, Department Woman-Mother-Child, Lausanne University Hospital and University of Lausanne, Lausanne, Switzerland; 2 The Radiology Department, Lausanne University Hospital and University of Lausanne, Lausanne, Switzerland; 3 The Sense Innovation and Research Center, Lausanne and Sion, Switzerland; 4 Laboratory for Neurorehabilitation Science, Medicine Section, Faculty of Science and Medicine, University of Fribourg, Fribourg, Switzerland; 5 Service of Endocrinology, Diabetes and Metabolism, Lausanne University Hospital and University of Lausanne, Lausanne, Switzerland; 6 Dartmouth College Thayer School of Engineering, Hanover, New Hampshire, USA; 7 Department of Surgery, Geisel School of Medicine, Dartmouth College, Hanover, New Hampshire, USA; 8 Faculty of Biology and Medicine, University of Lausanne, Lausanne, Switzerland; 9 Institute of Higher Education and Research in Healthcare, University of Lausanne, Lausanne, Switzerland; 10 Neonatology Service, Department Woman-Mother-Child, University Hospital of Lausanne, Lausanne, Switzerland; 11 Canadaian Women’s Heart Health Centre, University of Ottawa Heart Institute, Ottawa, Ontario, Canada; 12 School of Epidemiology and Public Health, University of Ottawa, Ottawa, Ontario, Canada

**Keywords:** Pregnancy, Electroencephalography, Obesity, Observational Study, Overweight

## Abstract

**Abstract:**

**Introduction:**

During pregnancy, physiological and psychological factors influence eating behaviour and food preferences. Food cravings are common in pregnancy and contribute to excessive gestational weight gain (GWG). Excessive GWG is present across all body mass index (BMI) categories and is associated with adverse outcomes. Outside of pregnancy, highly palatable food cues activate brain regions involved in reward and attention, which may influence eating behaviour and behavioural responses. However, brain and behavioural responses to food cues across pregnancy, and their association with psychological and metabolic factors, remain poorly understood. This study aims to investigate spatiotemporal brain responses to visual food cues across individuals with different BMI categories and their associations with behavioural, psychological and metabolic outcomes during pregnancy.

**Methods and analysis:**

This is a prospective observational cohort study conducted at the Lausanne University Hospital, Switzerland. 94 pregnant individuals (47 healthy normal weight and 47 with overweight/obesity) will be assessed at 12–16 and 31–36 weeks of gestational age from November 2024 to September 2026. Data collected for the primary outcomes include electroencephalography-based brain responses to validated visual food cues varying in fat and carbohydrate content, behavioural responses to the same cues using a gamified smartphone Go/NoGo task. Secondary validated outcomes include heart rate variability (Actiheart), body composition (InBody S10), glycated haemoglobin (Afinion), cardiorespiratory fitness (The Chester step test), snack intake, eating behaviour (Intuitive Eating Scale-2, Three-Factor Eating Questionnaire-Revised), food cravings (Food Craving Questionnaire-state), mood and depressive symptoms (Edinburgh Postnatal Depression Scale). Statistical analyses include group comparisons, longitudinal analyses and regression models adjusted for potential sociodemographic/medical confounders.

**Ethics and dissemination:**

All participants will provide written informed consent. The Human Research Ethics Committee of the Canton de Vaud approved the study protocol (CER-VD 2023-01462). Findings will be disseminated through peer-reviewed publications, (inter)national conferences and shared with healthcare professionals and stakeholders to inform strategies for improving maternal health.

**Strengths and limitations of this study**

- This study uses a multimodal approach to investigate pregnancy-related food cravings by combining self-reported with neurophysiological, behavioural psychological and physiological measures.
- Longitudinal design enables assessment of food cravings and brain responses to visual food cues across pregnancy.
- The relatively small sample size is consistent with comparable electroencephalography studies but may limit statistical power for subgroup or multivariable analyses.
- Due to limited availability of neurophysiological and behavioural data on food cue responses in pregnancy, several analyses are exploratory.
- The recruitment strategy and the requirement for multiple assessments may have introduced selection bias, potentially limiting the generalisability of the findings.

## Introduction

 Pregnancy is a period marked by many physiological and hormonal changes that are likely to influence eating habits, with an increase in appetite, food cravings and energy intake.^1^ Food cravings increase in frequency and intensity during pregnancy,^2^ with approximately 80% of pregnant women reporting food cravings at the end of the first trimester.^3^ They refer to strong urges for specific foods and are recognised as important contributors to overweight and obesity (OW/OB), excessive gestational weight gain (GWG) and failure to sustain weight loss in the postpartum period.^4^ The prevalence of excessive GWG for women of all prepregnancy body mass index (BMI) categories is between 37% and 64%.^5^ Excessive GWG is associated with long-term weight retention^6^ and increases the long-term risk of cardiometabolic morbidity and mortality.^7^ This is even more important in view of the globally rising prevalence of obesity that affects 35.7% of women within the reproductive age in the USA.^8^

Psychological states such as stress, mood and emotions may further influence eating behaviours, including the quality of food intake. Both stress and emotions have an impact on eating behaviour during pregnancy, including the increased risk of consuming food with higher energy density and less nutritional value.^9^ Similarly, negative mood states and higher levels of depressive symptoms and stress are associated with more frequent and intense self-reported reward-related eating and addictive-like eating and food cravings.^10–12^

Food cravings might further be influenced by regular physical activity. In the general population, an association between regular moderate-to-vigorous physical activity and reduced wanting for high-fat food was found.^13^ In pregnancy, physical activity showed benefits for preventing adverse pregnancy outcomes, such as excessive GWG or gestational diabetes mellitus (GDM).^14^ A cohort of women followed from their first to third trimester of pregnancy with normal weight (NW) or with obesity found high physical activity levels to be associated with higher levels of circulating soluble leptin receptor in both the second and third trimester, suggesting a potential association between exercise, reward-related pathways and neuroendocrine regulation of food intake.^15–17^

Neurobiological research in rodents demonstrates that pregnancy induces a reorganisation of key components of the dopaminergic mesolimbic circuitry promoting craving-like behaviour and an increased intake of palatable foods.^18^ However, to our knowledge, there is no clear understanding of the brain mechanisms underlying food cravings during pregnancy in humans. Highly palatable foods, such as those high in sugar or fat, are known to trigger the reward system and promote overeating.^19^ A study found that self-reported cravings for high-fat foods triggered by emotional cues are particularly linked to excessive GWG.^20^ Such palatable foods activate limbic and striatal reward pathways, increasing the tendency to seek and consume them.^21^ Speed of motor responses in discrimination tasks, such as Go/NoGo, may reflect the reward system’s sensitivity to food cues. The more motivational value a cue has, the faster the motor response.^22^ However, there is a lack of knowledge regarding behavioural and brain responses to food cues during pregnancy in humans.

Research outside of pregnancy has shown that the human brain can discriminate the energy value of foods when viewing food images, particularly fat content. Cues of ‘high’ versus ‘low’ fat foods elicit differential brain responses in both strength and topography, especially in prefrontal cortical regions involved in reward evaluation and decision-making.^23^ Other neuroimaging studies have shown that the dorsolateral prefrontal cortex plays a key role in cognitive control of hunger, appetite, and reward evaluation, and reduced activation in the region has been associated with obesity and poorer appetite regulation.^24 25^ Furthermore, the brain’s response to food stimuli differs between women with different weight categories: women with overweight showed increased visual evoked potentials (VEPs) in the right frontal and central cortices, which are markers of an increased sensory alertness and a heightened visual cue-reactivity, not allowing to downregulate later responsivity.^26^

The above-mentioned research on food cravings during pregnancy has been based on questionnaires assessing different types of food cravings and their occurrences.^10–12 19 20^ However, no study has yet identified any underlying mechanisms to objectively describe or explain the cause of cravings during pregnancy.

## Aims

The overall aim of this study is to investigate brain responses to food viewing in the beginning of the second and the third trimesters of pregnancy and their relationship with metabolic health. We will investigate this in pregnant individuals with NW and with OW/OB, which are at higher metabolic risk.

- Primary objectives:
  - To investigate differences in brain and behavioural responses to food pictures of varying fat and carbohydrate content: high-fat/high-carbohydrate (HFHC), high-fat/low-carbohydrate (HFLC), low-fat/high-carbohydrate (LFHC) and low-fat/low-carbohydrate (LFLC) during pregnancy, in women with and without OW/OB.
  - To investigate the relationships between brain responses to food pictures as measured by electroencephalography (EEG) and the behavioural responses to food cues measured with a smartphone-based Go/NoGo task in these individuals.
- Secondary objectives in the same population:
  - To determine the relationships between brain responses to food pictures and different emotional states and stress levels.
  - To investigate differences in brain and behavioural responses to food and non-food pictures.
  - To investigate longitudinal changes in brain and behavioural responses to food pictures of varying fat and carbohydrate content from early to late pregnancy.
  - To investigate the cross-sectional and longitudinal relationships between brain responses to food viewing with eating behaviour, metabolic health and mental health during pregnancy.

## Methods

### Study design

**Figure 1:**
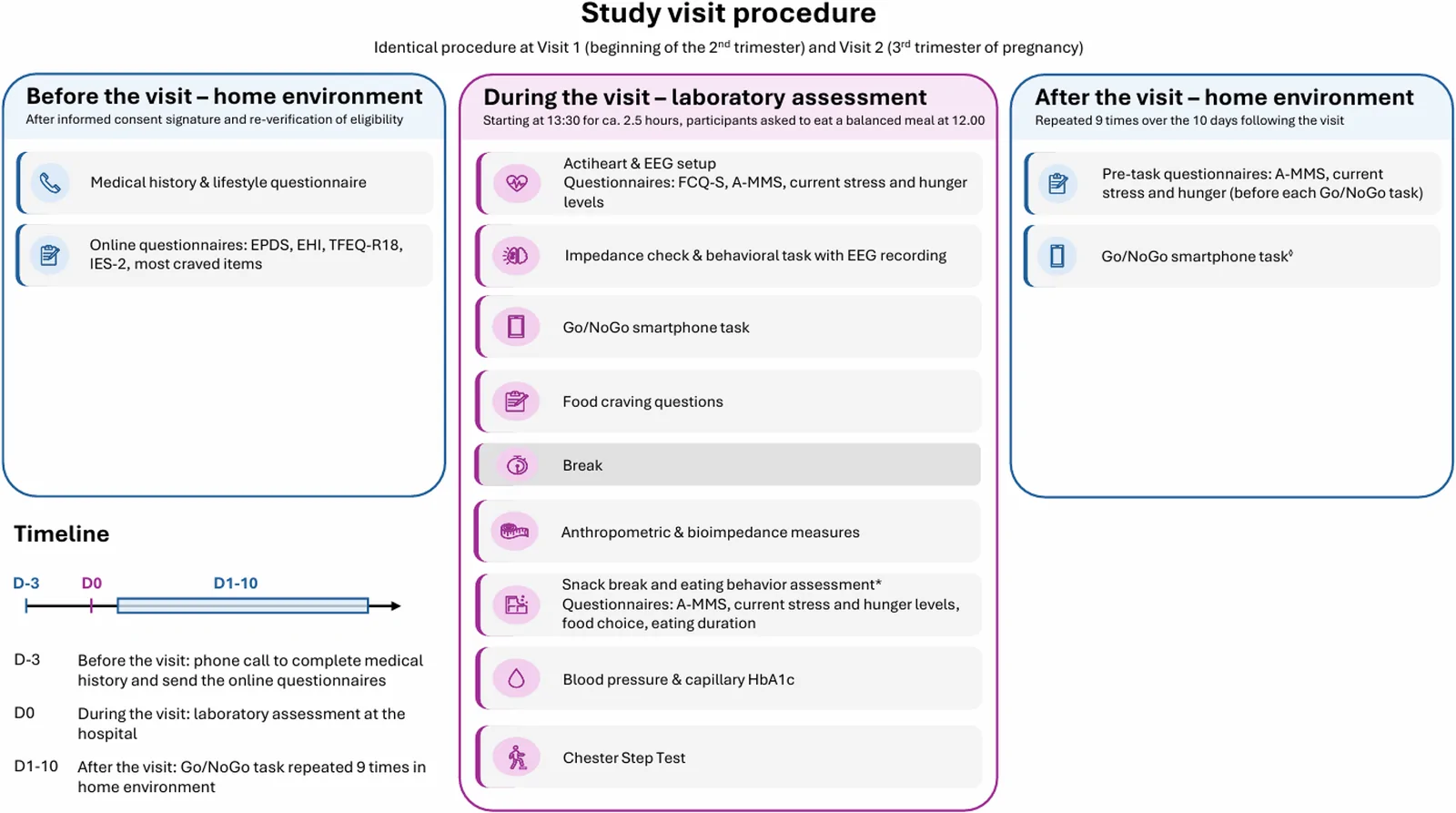
Flow chart of the study procedure. *During the snack break participants’ eating behaviour will be recorded with an objective eating behaviour recognition device while participants can choose to consume any of the eight provided snacks. ◊3 days in the morning awakening, 3 days in the evening before going to sleep as well as three times when specifically stressed or in a particularly aroused mood during 10 days after each visit. A-MMS, adapted version of the Multidimensional Mood Scale; EEG, electroencephalography; EHI, Edinburgh Handedness Inventory; EPDS, Edinburgh Postnatal Depression Scale; FCQ-S, Food Cravings Questionnaire-State; HbA1c, glycated haemoglobin; IES-2, Intuitive Eating Scale-2; TFEQ-R18, Three-Factor Eating Questionnaire-Revised 18.

This is a prospective observational study of pregnant individuals with and without OW/OB. Participants will complete two study visits at the Lausanne University Hospital during pregnancy: one during the second trimester (12–16 weeks gestational age (GA)) and the second visit during their third trimester (31–36 weeks GA, see figure 1 for details).

### Study population and eligibility criteria

A total of 94 participants with valid data for the primary outcomes will be included. The study will include two groups of participants based on their prepregnancy BMI, one including individuals with NW and a second group of individuals with OW/OB. Recruitment will be done through flyers in the Lausanne University Hospital and various ambulatory regional hospital clinics as well as private obstetrics and gynaecology practices in the canton of Vaud. Eligibility will be determined during recruitment using a screening questionnaire before enrolment. Participants will self-report all inclusion and exclusion criteria. For both groups, inclusion criteria will include pregnant women with GA between 12 and 16 weeks, aged ≥18 years, and willingness and ability to provide signed informed consent. Additional inclusion criteria for the NW group will be: prepregnancy BMI <25 kg/m^2^, no restrictive diets during pregnancy, no weight changes exceeding 5 kg in the past 5 years (excluding pregnancy), and weight gain within 2 kg during the first trimester, in accordance with the National Academy of Medicine recommendations at the time of inclusion.^27^ Inclusion criteria for individuals in the OW/OB group will be prepregnancy BMI ≥25 kg/m^2^.

Exclusion criteria will include pre-existing diabetes, a diagnosis of an eating disorder (eg, anorexia nervosa, bulimia nervosa or binge-eating disorder), vegetarian or vegan diet (to ensure familiarity and exposure to all food categories), uncontrollable nausea and vomiting during the first trimester of pregnancy, history of bariatric surgery, participation in an intervention study, diagnosed mental illness or use of medication to treat psychiatric disorders, as well as active suicidal thoughts and having an implanted cardioverter-defibrillator.

### Primary outcomes

The primary outcomes of this study include EEG brain responses to food categories of varying fat and carbohydrate content (HFHC, HFLC, LFHC and LFLC), characterised by the VEP latency, amplitude, duration, topography and neural source activity localisation. Additional primary outcomes include the speed and accuracy of the behavioural responses to food categories of varying fat and carbohydrate content (HFLC; HFHC; LFLC; LFHC) measured by a Go/NoGo smartphone task.

### Secondary outcomes

The secondary outcomes include weight, glycated haemoglobin (HbA1c) and body composition. We will also investigate eating behaviour through self-reported questionnaires including food cravings (Food Craving Questionnaire-state, FCQ-s), intuitive eating (French Intuitive Eating Scale-2, IES-2), external, emotional eating and cognitive restriction (Three-Factor Eating Questionnaire-Revised 18, TFEQ-R18). We will measure eating rate using an objective eating behaviour recognition device and subjective questions (self-reported eating rate). We will measure the current mood (Adapted Multidimensional Mood Scale, A-MMS), current level of perceived stress (single-item) and depressive symptoms (Edinburgh Postnatal Depression Scale, EPDS). We will measure heart rate (HR) and heart rate variability (HRV) using an Actiheart. We will finally investigate aerobic fitness using the Chester Step Test (CST).

### Data collection and study visits

The first participant attended the first visit in November 2023 at the Lausanne University Hospital. The last participant is expected to be recruited in May 2026, and all data are expected to be collected by September 2026. The first visit will take place between 12 and 16 weeks of GA, and the second visit will take place between 31 and 36 weeks of GA.

After signing the informed consent, a first phone call will be scheduled, where participants will be asked to provide information about their medical history and lifestyle behaviour. At the end of the phone call, participants will fill out an online questionnaire, including several validated questionnaires (figure 1).

Before each visit, participants will be asked to eat a balanced meal around 12:00 (based on the Swiss Society for Nutrition recommendations, containing a source of carbs, proteins and fibres and avoid fast food or sugary beverages) to minimise the bias of different satiety levels and will be invited to come to the Lausanne University Hospital at 13:30. The testing will be completed at 16:00 and will include several breaks (figure 1).

### Patient and public involvement

Individuals participating in our previous study^28^ expressed a high level of interest in the use of EEG as an objective measure for neurobehavioural responses during pregnancy. A subset of participants (n=16) was asked in semistructured interviews to cite the most meaningful aspects to research in the perinatal period. Based on thematic analyses, the most important research areas for the 16 women were GWG (n=14), nutrition in the perinatal period (n=14), eating behaviour (n=8) and cravings (n=5). Further interests reported by participants included postpartum weight retention and emotional changes during pregnancy. This feedback shaped our present study and primary research questions to gain knowledge on objective measures for food cravings and eating behaviour during pregnancy and their impact on mental and metabolic health.

### Measures

Except for sociodemographic data, all measures will be collected at both visits (see figure 1 for details).

#### Food categories

Across the entire study, both the food pictures and food items are classified into four categories based on their fat and carbohydrate content. The categories are HFHC, HFLC, LFHC and LFLC. Foods were classified as high-fat (>5 g of fat per 100 g (21.6±13.77 g/100 g)) or low-fat (<5 g of fat per 100 g (1.1±1.29 g/100 g)) and as high-carbohydrate (>15 g of carbohydrates per 100 g (48.57±21.13 g/100 g)) or low-carbohydrate (<15 g of carbohydrates per 100 g (4.59±4.59 g/100 g)).

#### EEG: stimuli and apparatus

Participants will be presented with four blocks of trials containing a total of 240 pictures. Each block will contain 50 food pictures of the four food categories and 10 non-food objects that were selected from a larger food picture database.^29^ All pictures will be standardised in size and controlled for low-level visual properties.^23 30^ Each picture will be presented for 500 ms, with block order randomised across participants. A central fixation cross will be displayed during interstimulus intervals to minimise eye movements. The experimental session will take place in a sound-attenuated booth (WhisperRoom model 102 126E). Visual stimuli will be presented on a 27-inch LCD monitor (HP EliteDisplay E273q, 2560×1440 pixels, 60 Hz). Continuous EEG data will be recorded at 1000 Hz using a 128-channel gel-based electrode cap connected to an EEGO mylab system (ANT Neuro; https://www.ant-neuro.com). The online reference will be an electrode at the vertex. Electrodes in the cap are spaced equidistant to one another.

#### Behavioural task during EEG recording

Participants will complete a speeded two-alternative forced choice discrimination task across the four previously described blocks of trials, lasting approximately 13 min in total. During this task, participants will indicate whether each cue presented represents a food or a non-food item by pressing a button on a response box with their dominant hand (button ‘1’ for food pictures, button ‘2’ for non-food object pictures). The block order will be randomised across participants.

The experimenter will first provide a detailed explanation of the task, followed by a brief practice session with seven food and three non-food pictures to familiarise participants with the procedure. Additionally, short on-screen instructions will be presented at the start of each block. Participants will be encouraged to take brief breaks between blocks to reduce fatigue.

Behavioural measures will include the mean response time (RT) (in milliseconds) for correctly identified food and non-food pictures, as well as for each food category. Accuracy (%) will be calculated as the proportion of correctly responded trials. The same selection of food cues will be shown on both visits.

#### Handedness

The handedness of each participant is calculated using the 4-item Edinburgh Handedness Inventory–Short Form.^31^ This is a validated short form of the original questionnaire^32^ where participants indicate their preferred hand for four activities on a 5-point Likert scale from ‘always right’ to ‘always left’. Responses will be recorded from –2 ‘always left’ to +2 ‘always right’ and summed to compute a total handedness score. Participants will be classified as right-handed if total >0, left-handed if total <0 or ambidextrous if total=0.

#### Go/NoGo gamified task

A specific smartphone will be provided to complete a gamified Go/NoGo task lasting a total of approximately 15 min. Participants will be asked to complete this task 10 times in total; once during each of the visits and nine additional times per visit at home. This task will include 24 randomised food cues of varying fat and carbohydrate content (see section ‘Food categories’) circled in green (Go cues), and 4 randomised object cues circled in red (NoGo cues). In total, there will be 70% of Go cues and 30% of NoGo cues. Participants will complete multiple runs of the task each day until they fulfil a daily objective, receiving intermediate scores after each run, and a final score when the objective is achieved.

Before the first game, participants will be asked in the app to answer a liking questionnaire (Visual Analogue Scale (VAS) from 0=strongly dislike to 100=strongly like), where they will rate food images presented during the Go/NoGo task. Before each task, participants will be asked to answer a questionnaire regarding their current mood (A-MMS), current level of perceived stress (single item) and current level of hunger (single item).

During the task, participants will be instructed to respond as fast as possible to the food cues (Go trials) by sliding the image downward with their finger, while withholding their responses to the non-food cues (NoGo trials). If participants respond to a food cue on time (Hit) or correctly withhold their response to an object cue (correct rejection, CR), positive green feedback will be displayed. If participants fail to respond to a food cue within 1.5 s (Miss) or respond to an object cue (false alarm, FA), negative red feedback will be displayed.

As part of the task’s gamified design, and to maintain engagement and adherence, participants who will correctly respond to food cues but exceed the reaction time threshold (RTT, 725 ms) will receive a negative ‘Late’ feedback, while a ‘Down’ feedback will be displayed if the food cues are not dragged to the bottom of the screen.

The behavioural output of the gamified Go/NoGo task will include the response type (Hit, CR, FA, Miss), the feedback (Correct, Incorrect, Late, Down), and the individual reaction time (RT) in milliseconds to each food cue. The outcomes will then be analysed in the percentage of successful trials, total number of errors (FA or missed food cue), number of late (Hit with a RT above the RTT), the mean RT (ms) to food cues, mean RT to each food category (HFLC; HFHC; LFLC; LFHC), total mean RT for food cues and inhibition error (proportion of FA relative to the total of FA and CR).

#### HR and HRV

HRV, which is influenced by stress, reflects the autonomic nervous system’s level of arousal and can serve as an objective measure of psychological well-being and experienced stress.^33^ HR and HRV will be continuously recorded throughout the visits using the Actiheart device (CamNtech, Papworth, UK). Actiheart is a validated instrument that reliably measures HR and interbeat interval (IBI).^34^ We will use the Actiheart model 4 that consists of two monitors which will be placed on the midsternal line and on the left thorax on the level of the third intercostal space.

To ensure stable baseline and recovery measurements, participants will be asked to remain seated in a resting position for 2 min before and after the EEG recording and the snack. The duration will be timed using a stopwatch to standardise measurement periods.

The commercial Actiheart Software Kubios HRV Premium (V.3.5.0) software^35^ will be used for data cleaning and transformation to export IBI. Kubios is a software for HRV analysis and computes time-domain and frequency-domain parameters.^36^ Time-domain parameters are calculated from the IBI and inform about variability between successive R-peaks. The frequency domain measures estimate the distribution of absolute and relative power into four frequency bands. Time-domain variables to be included are mean HR, the root mean square of successive differences between normal heartbeats, and the percentage of successive RR intervals that differ by more than 50 ms (pNN50). For frequency-domain variables, we will include high-frequency (HF) and low-frequency (LF), both in absolute units (ms^2^) and normalised units, as well as the LFHF ratio.

#### Anthropometric measurements and body composition

Participants’ body weight and height will be measured using a regularly calibrated electronic scale (Seca Model 7017021094, Hamburg, Germany). Body composition will be measured using bioelectrical impedance analysis (BIA). Low-voltage electrical currents will be applied through electrodes placed on the extremities. The BIA 101 (Akern, Italy, medically certified) measures tissue resistance and reactance using four electrodes: two on the right hand and two on the right foot, separated by 3–4 cm. The InBody S10 system is based on a 4-compartment model, estimating total body water, protein, mineral and body fat and uses electrodes on all four extremities. Measures of interest will include fat mass, fat-free mass, visceral fat surface and the percentage of these measures related to body weight as well as calculated waist circumference and phase angle.

#### Snack and objective eating behaviour

During both study visits, participants will be offered eight snacks of different textures and levels of crunchiness during a snack break. The snacks vary in fat and carbohydrate content with two snacks from each food category described above. At both visits, participants will be presented with potato chips (10 g), milk chocolate (25 g), peanuts (20 g), almonds (20 g), pretzels (15 g), gummy bears (25 g), tomatoes (50 g) and blueberries (50 g) corresponding to one portion of each snack item. All snacks are safe and appropriate for pregnancy and fresh fruits and vegetables will be thoroughly washed and prepared according to standard pregnancy food-safety guidelines. Participants will be informed that they can eat as much or as little as they want, with no expectations regarding completion. The type and quantity of snacks consumed, as well as the time spent eating, will be recorded by the study team. Once participants indicate they have finished eating, they will be asked regarding their perceived eating duration and eating speed.

Objective eating behaviour will be assessed using an eating behaviour recognition device, which uses an accelerometer (ADXL362) and contact microphone (CM-01B) positioned over the mastoid bone to detect chewing-related motion and vibrations transmitted through the mandible during eating. During the standardised snack, the device will continuously record chewing-related signals to derive objective eating behaviour measures, including eating onset latency (seconds), eating duration (minutes), eating rate (event/time) and other temporal characteristics of eating behaviour. These objective measures will complement the observed snack intake (type and quantity consumed) and participants’ self-reported eating speed and eating duration. They will be used to investigate whether objectively measured eating behaviour is associated with neural responses to visual food cues measured by EEG and behavioural responses measured by the Go/NoGo task, and whether these associations differ between women with NW and those with OW/OB. This eating behaviour recognition device has previously demonstrated high accuracy for automated eating detection using chewing-related motion and vibration signals.^37^

#### Glycated haemoglobin

HbA1c levels (%) will be measured for each participant at both study visits from a capillary blood sample using the validated Afinion 2 point-of-care analyser.^38^ This device uses a chemical photometric method with boronate conjugation.^39^ The measure represents the mean glucose levels over the last 2–3 months and will be used as a continuous variable.

#### Chester step test

The CST will be used to estimate the participants’ cardiorespiratory fitness (VO_2_max indicated in mL/kg/min). The CST is a multistage submaximal test that has demonstrated reasonable validity against indirect calorimetry to predict cardiorespiratory capacity.^40^ From the CST results, a standardised equation will be used to estimate the VO_2_max.^40^ After a 30 s familiarisation, participants will step up and down to an audiotape starting at 15 steps/min, increasing by five steps/min every 2 min, for a maximum of 10 min. The step height will be set at 15 cm. HR and rate of perceived exertion (RPE, according to Borg’s scale) will be monitored at the end of every 2-minute stage. The test will be stopped at 80% of the estimated HRmax (220 – age), RPE ≥15, or when participants show signs of distress.

#### Eating behaviour and hunger

Eating behaviour will be assessed using the French IES-2. This is a validated self-reported questionnaire that assesses participants’ tendency to rely on physical hunger and satiety cues and has demonstrated good psychometric properties.^41 42^ The IES-2 consists of 18 items across 3 subscales: Reliance on Hunger and Satiety Cues (RHSC, six items), Eating for Physical rather than Emotional Reasons (EPR, eight items) and Unconditional Permission to Eat (UPE, four items). Items are scored on a 5-point Likert from 1=‘strongly agree’ to 5=‘strongly disagree’, with subscale scores ranging from 1 to 5. For the RHSC subscale, higher scores indicate greater trust in internal hunger and fullness signals, while lower scores indicate reduced capacity to regulate food intake based on these cues. For the EPR subscale, higher scores indicate eating in response to physical hunger, whereas lower scores reflect eating for emotional reasons. For the UPE subscale, higher scores indicate unconditional permission to eat, whereas lower scores indicate restrictive eating.

Three dimensions of eating behaviour will be assessed using the TFEQ-R18. These dimensions include cognitive restraint (six items), uncontrolled eating (nine items) and emotional eating (three items). Items will be scored on a 4-point Likert scale ranging from 1=‘almost always’ to 4=’never’. Raw scores will be calculated for each subscale and then transformed into percentage scores, representing the relative proportion of the highest possible raw scores (0 to 100). The revised questionnaire has been shown to have good psychometric properties.^43^

Current food cravings will be assessed through the FCQ-s which will evaluate the intensity and presence of cravings at a given time in response to specific situations or emotional and physiological cues. The 15 items will be scored on a 5-point Likert scale ranging from 1=‘strongly disagree’ to 5=‘strongly agree’. The total score will represent the current food craving state, with higher scores indicating more intense current food cravings. The French version was translated and used in previous research by our collaborators.^44^

Current food ‘wanting’ will be assessed using the item ‘How much do you want this food?’ rated from 0 to 100 on a VAS. Participants will provide ratings for 24 food images of varying fat and carbohydrate content. This will take place immediately after the EEG recording.

Current hunger will be assessed with a single item ‘What is your current level of hunger?’ rated on a 0 to 100 VAS, with 0=‘not hungry at all’ and 100=‘very hungry’.

#### Mental health, mood, and stress

Depressive symptoms during pregnancy will be assessed using the EPDS, a validated 10-item self-reported questionnaire. Each item will be rated on a 4-point Likert scale with higher scores indicating greater depressive symptoms, and the total scores range from 0 to 30 points. This is a validated self-reported questionnaire that demonstrates good psychometric properties in the perinatal period.^45^

Current mood will be assessed using an adapted version of the Multidimensional Mood Scale, consisting of eight bipolar items.^46 47^ Each item will be presented on a 0 to 100 bipolar scale between opposing mood states (eg, tired vs awake), with higher values indicating a mood closer to the right-hand pole. Responses will be provided with a slider from the start position at 50. In addition, the feelings of fear, joy, sadness, and anger were assessed using a VAS (from 0 to 100).

Current level of perceived stress will be assessed with a single item ‘What is your current level of stress?’ rated on a 0 to 100 VAS with 0=‘not stressed at all’ and 100=‘very stressed’.

### Data management and statistical analyses

#### Sample size calculation

The sample size estimation was informed by previous EEG studies of similar design outside of the perinatal context,^23 28 48^ as well as internal pilot data from our group. For EEG outcomes, preliminary data from our previous study (CER-VD 2021-01976) showed a significant fat x group interaction in global field power (GFP) (ηp²=0.103, F(1,37)=4.237, p=0.047; Cohen’s f=0.34). Power analysis assuming an alpha level of 0.05 and 80% power indicated that 35 participants per group were needed. For the Go/NoGo smartphone task used in a different context, pilot data demonstrated group differences in RTs of 550 ms±63 in women with NW and 450 ms±63 in women with OW/OB, at least eight participants per group would be required to detect significant differences in RT assuming an alpha level of 0.05 and 80% power.

Participants diagnosed with GDM at the second visit (approximately 16%) will be excluded in sensitivity analyses. To account for these exclusions and an additional anticipated dropout of 10%–20%, a total of 94 participants (47 per group) with valid data will be recruited. This is expected to result in approximately 37 participants per group at the second visit with valid data, corresponding to the highest requirement derived from our calculations.

#### Statistical analysis plan

Statistical analyses will be performed with R and SPSS software. Demographic, behavioural and other descriptive variables will be represented as means and SDs or percentages where appropriate. Analysis of variance, t-tests or χ^2^ tests will be used to determine differences between groups (in brain responses, eg, differences between responses to food and non-food pictures, differences in responses between different food categories, as well as differences in brain responses between the second and third trimester of pregnancy and between NW women and OW/OB). Paired t-test will be used to determine within-subject differences between both timepoints (second and third trimester). Regression analyses will be used to model the relationship between predictor variables and outcomes (such as between brain responses to food viewing with intuitive eating and metabolic health). Associations between objective eating behaviour measures and neurobehavioural responses to food cues will be evaluated using correlation and multivariable regression analyses. Specifically, we will examine whether eating onset latency, eating duration and eating rate are associated with EEG responses to food images and behavioural performance on the Go/NoGo task, and whether these relationships differ according to the BMI group. We will also investigate the impact of covariates such as maternal age, GA, prepregnancy BMI on outcomes and adjust for them when necessary.

##### Processing of the EEG data

The preprocessing of the EEG recordings will be conducted in Cartool Freeware^49^ and will include band-pass filtering (0.1 Hz high-pass and 40 Hz low-pass) and a 50 Hz notch filter, both using a fourth-order non-causal bidirectional Butterworth filter (−24 dB/octave roll-off). Epochs from –100 to 500 ms relative to stimulus onset will be extracted and averaged for each participant and food category to derive the VEPs. Trials will be rejected when voltages at any channel exceed ±100 μV (automated artefact rejection criteria) or visual controls for artefacts (eg, blinks, eye movement). Likewise, channels with poor signal will be excluded. Electrodes will be interpolated using 3-D splines individually for each participant before averaging the data.^50^ Data will then be baseline corrected to the –100 ms prestimulus period and recalculated against the common average reference. After preprocessing, participants with less than 60% of included trials per food category will be excluded from the analyses.

##### VEPs analyses

Differences in brain responses to the stimuli between groups will be assessed using an electrical neuroimaging framework in a multistep analysis procedure that will include both global and local electric field measures.^51 52^

##### Topographic clustering

A topographical clustering analysis will be conducted using hierarchical clustering in Cartool over the 500 ms post-stimulus group-averaged VEPs. This analysis will identify periods of stable scalp electric field topographies (‘template maps’). This analysis is insensitive to differences in response amplitude across the four categories, as data are first normalised by instantaneous GFP. The optimal number of template maps will be determined using the meta-criterion.^53^ A fitting procedure will then assign each time point of each participant’s VEP to the template map that presented the strongest spatial correlation.^54^ This fitting quantifies the preponderance of each template map over a specific time window, which will be analysed using mixed-model repeated measures analysis of variance (rmANOVA). Since topographic differences follow from changes in the underlying source configuration, we will be able to discern whether and when intracranial generator configurations differ between food categories and/or groups.^51^

##### Global field power

To quantify the differences between groups and categories in strength of the VEPs, GFP will be calculated for each participant and food category. GFP is the spatial SD of the electric field of all electrodes and is used to quantify response strength at each time point, considering data from all electrodes simultaneously.^55^ Mean GFP within each time window, based on the abovementioned topographic clustering analysis, will be analysed using rmANOVAs with fat and carbohydrate as within-subject factors and group as the between-subject factors. Significant interactions will be adjusted for multiple comparisons. These analyses will be performed using SPSS.

##### Source estimations

Intracranial sources of the VEPs for the four food categories will be estimated using a distributed linear inverse solution based on the Local Autoregressive Average Regularisation Approach (LAURA)^56^ as implemented in Cartool. The head model and lead-field matrix will be computed using the Spherical Model with Anatomical Constraints,^57^ providing current density measures as output, with scalar values at each of 3058 nodes in the solution space. VEPs will be averaged across the predefined time windows (from the topographic clustering) for each participant and food category, and source activity will be computed at each node. RmANOVAs will be conducted using STEN toolbox version 2.0 developed by Jean-François Knebel and Michael Notter (http://doi.org/10.5281/zenodo.1164038) to compare source activity between groups and conditions. Differences in source activity will be considered significant when an activity differed as a function of group and/or condition at the level of individual nodes (p<0.05) and clusters of at least 10 contiguous nodes survive the statistical threshold form a reliable cluster.^48 58^ Post hoc paired and independent sample t-tests with adjustment for multiple comparisons will be performed in SPSS to further investigate within-group and between-group differences.

##### Analysis of the gamified Go/NoGo task

Raw data from the gamified Go/NoGo task will be collected through The Diner smartphone application and preprocessed using the Diner Data Processor, a Matlab-based processor. For each participant, all raw data files will be extracted directly from the smartphone.

Raw log files will then be analysed and transformed into an Excel dataset for each participant through the processor.

Behavioural data will be imported from Excel files to R running or RStudio will be used^59^ and packages from tidyverse.^60^ Mean RTs will be calculated from the correct responses (Hit trials) for each food item, food category, participant and training day. The food category mean RTs will be calculated by averaging the RT of all items within each food category for each participant and day. Overall RT to food cues will be computed by averaging across all food categories, participants and day. In addition, accuracy measures, including the percentage of correct responses, number of errors, and inhibition error rates (FA/CR+FA)*100, will be computed per participant and per day. Further analyses will include modelling using repeated measures analyses of each day.

## Ethics and dissemination

The study has been approved by the Human Research Ethics Committee of the Canton de Vaud (CER-VD 2023-01462). Written informed consent will be obtained for all participants before any study data are collected. With explicit participant consent, personal medical data (body weight, BMI, body composition, blood pressure, HbA1c) collected during the study visits will be communicated to the participants’ obstetrician or general practitioner in the form of a medical report at the end of their participation.

Once the formal analyses of this study are completed, participants will be invited to a presentation where these will be presented and discussed. Additionally, results will be disseminated by presentations at national and international conferences, publications in peer-reviewed scientific journals, and other scientific meetings.
