# Supplementary material for: Brain and behavioural responses to food viewing in women during pregnancy and their relationship with metabolic health: study protocol for the FOODY Play Study – a prospective observational study
Source: BMJ Open. 2026 Sep 11;16(9):e119379. doi: 10.1136/bmjopen-2026-119379 (PMC13583829; doi:10.1136/bmjopen-2026-119379)
Supplement: Supplementary file 1 [file bmjopen-2026-119379.reviewer_comments.pdf]

PEER REVIEW HISTORY

BMJ Open publishes all reviews undertaken for accepted manuscripts. Reviewers are asked to complete a checklist review form and are provided with free text boxes to elaborate on their assessment. These free text comments are reproduced below.

ARTICLE DETAILS

Title (Provisional)

Brain and behavioral responses to food viewing in women during pregnancy and their relationship with metabolic health: study protocol for the FOODY Play Study, a prospective observational study

Authors

Treviño Montemayor, Mariana; Hamam-Mechkour, Amel; Eisler, Jennifer J.; Murray, Micah; Spierer, Lucas; Schenk, Sybille; Halter, Ryan J; Toepel, Ulrike; Horsch, Antje; Retsa, Chrysa; Arhab, Amar; Quansah, Dan Yedu; Puder, Jardena J

VERSION 1 - REVIEW

Reviewer 1

Name Daneshvar, Mojtaba

Affiliation Tehran University of Medical Sciences, faculty of nutritional sciences and dietetics, Tehran University of Medical Sciences, Tehran, Iran

Date 17-Jun-2026

COI None

This is a well-written, innovative, and methodologically sound study protocol. The use of an objective, gamified Go/NoGo task in a smartphone app and the objective eating behavior recognition device are particularly forward-thinking and address a key gap in the literature. The inclusion of a PPI component significantly strengthens the research question's relevance. I believe this protocol is suitable for publication in BMJ Open following minor revisions.

Major Comments

None.

Minor Comments

1) Trial Registration: It is a standard requirement for trial studies to be registered in a publicly accessible registry. The manuscript does not include a trial registration number. I strongly

recommend that the authors register their study and include the registration number in the abstract and the main text.

2) Objective Eating Behavior: The protocol states that the device will measure "eating rate, rapidity of eating onset, and other eating-related behavior." Please specify the precise units of measurement and clarify how these objective measures will be used to test the main hypotheses (e.g., do you hypothesize that faster eating rates in OW/OB women will correlate with heightened EEG responses to high-fat/high-carbohydrate cues?). Expressing the exact utility of these data within your main hypotheses will help readers appreciate the full value of this innovative measure.

3) Study Limitations: While the limitations are discussed adequately, adding a brief mention of the potential for "selection bias" would be prudent. As participants are a self-selected group willing to undergo these intensive assessments, they may not be representative of the general pregnant population. This could impact the generalizability of the findings.

regard

**Reviewer**	**2**
**Name**	**Aji, Arif Sabta**
**Affiliation**	**National Research and Innovation Agency Republic of Indonesia, Research Center for Biomedical**
**Date**	**06-Jul-2026**
**COI**	**None**

Dear Author,

We suggests the comment and recommendation in the pdf text.

**** **The reviewer provided a marked copy with additional comments. Please contact the publisher for full details.** ****

VERSION 1 - AUTHOR RESPONSE

Reviewer: 1
Dr. Mojtaba Daneshvar, Tehran University of Medical Sciences
Comments to the Author:
This is a well-written, innovative, and methodologically sound study protocol. The use of an objective, gamified Go/NoGo task in a smartphone app and the objective eating behavior recognition device are particularly forward-thinking and address a key gap in the literature. The inclusion of a PPI component significantly strengthens the research question's relevance. I believe this protocol is suitable for publication in BMJ Open

following minor revisions.

Major Comments
None.

Minor Comments
1) Trial Registration: It is a standard requirement for trial studies to be registered in a publicly accessible registry. The manuscript does not include a trial registration number. I strongly recommend that the authors register their study and include the registration number in the abstract and the main text.

> *We thank the reviewer for this valuable comment. We agree that prospective registration of research protocols can enhance transparency and reproducibility. However, our study is an observational study rather than an interventional clinical trial, and therefore trial registration was not a requirement. As the study was not prospectively registered in a public repository, no registration number is available to report. This study protocol publication would therefore provide all information regarding study design and methodology.*

2) Objective Eating Behavior: The protocol states that the device will measure "eating rate, rapidity of eating onset, and other eating-related behavior." Please specify the precise units of measurement and clarify how these objective measures will be used to test the main hypotheses (e.g., do you hypothesize that faster eating rates in OW/OB women will correlate with heightened EEG responses to high-fat/high-carbohydrate cues?). Expressing the exact utility of these data within your main hypotheses will help readers appreciate the full value of this innovative measure.

> *We thank the reviewer for this helpful suggestion. We have revised the manuscript to better describe the objective eating behavior outcomes derived from the Auracle™ device and their corresponding units of measurement. Specifically, we now specify the objective measures collected during the standardized snack (e.g., eating onset latency [seconds], eating duration [minutes], and eating rate (events/time). We have also clarified the role of these measures within the study hypotheses and statistical analysis. Specifically, objective eating behavior will be examined as a behavioral correlate of food cue reactivity to determine whether participants with greater neural and behavioral responsiveness to highly palatable food cues also demonstrate faster objectively measured eating behavior, and whether these relationships differ between women with normal weight and those with overweight or obesity.*

3) Study Limitations: While the limitations are discussed adequately, adding a brief

mention of the potential for "selection bias" would be prudent. As participants are a self-selected group willing to undergo these intensive assessments, they may not be representative of the general pregnant population. This could impact the generalizability of the findings.

We appreciate the reviewer's comment. We have revised the "strengths and limitations" section taking in accordance with this, the editor's and the other reviewers' comment. Specifically, we added a statement acknowledging the potential of selection bias resulting from the recruitment strategy and repeated assessments required for participation. We also note that this might limit the generalizability of the findings to the broader pregnant population.

Reviewer: 2
Dr. Arif Sabta Aji, National Research and Innovation Agency Republic of Indonesia
Comments to the Author:
Dear Author,

We suggests the comment and recommendation in the pdf text.

Abstract:

1) there is no information for the study setting, time, and location.
   We thank the reviewer for this comment. We have revised the abstract to include the study setting, period, and location to provide greater context of the study.

2) there is no clear a study design mentioned in this section. is this cohort study or case-control study?
   We thank the reviewer for this observation. The abstract has been revised to explicitly state the study design.

3) What the data of author mean? write in detail what kind of data that will be collected during the study.
   We appreciate the reviewer's comment. The abstract has been revised to provide a clear description of the data collected during the study.

4) Justify these instrument or tools if these are validated by the author or use the secondary data resource.
   We thank the reviewer for this suggestion. The abstract has been revised to include information on the instruments used.

5) add the analysis method and adjustment of this study written.
   We thank the reviewer for this suggestion. We have now included a more detailed statistical analysis section, including the adjustments used in the study.

6) add the dissemination that can beneficial to the stakeholders.

We thank the reviewer for this comment. The abstract has been revised to provide a statement on the dissemination of the study findings and their potential relevance and benefits to key stakeholders.

Strenghts and Limitations:

1) add one more strength or limitation of the study as the minimum number is four points.

We thank the reviewer for this comment. We have revised the "strengths and limitations" section taking in accordance with this, the editor's and the other reviewers' comment. Specifically, we added the longitudinal study design as a strength and as well as the potential of selection bias resulting from the recruitment strategy and repeated assessments.

Methods:

1) explain on how author determines the nutritional status of participants.

We thank the reviewer for this comment. We have clarified in the Methods section "Study population and eligibility criteria" how participants' nutritional status will be determined, including the criteria used to classify participants into the normal-weight and overweight/obesity groups.

2) how authors will diagnosis these exclusion criteria?

We appreciate the reviewer's comment. We have now clarified in the Methods section "Study population and eligibility criteria" that the exclusion criteria will be assessed during recruitment prior to study enrollment.

3) explain these acronyms first.

We thank the reviewer for this comment. We have now defined all acronyms at their first occurrence in the Methods section.

4) how to minimize the selection and measuring bias in this study?

We thank the reviewer for this comment. We have now addressed it in the Limitations section. Specifically, we noted that the recruitment strategy and the requirement for multiple assessments may have introduced selection bias, potentially limiting the generalizability of the findings.

5) explain how the authors could determine the glycated haemoglobin status or categorize.

We appreciate the reviewer's valuable comment. To improve clarity, we have added that HbA1c will only be used as a continuous variable. As such, participants will not be categorized using their HbA1c values.

Statistical analysis plan:

1) convert and present in the table, please.

We thank the reviewer for this comment. However, we are uncertain about the specific aspect of the statistical analysis plan that the reviewer is referring to. We would be grateful if the reviewer could clarify which information of the statistical analysis plan they found unclear. This will allow us to address the comment more accurately and effectively.

Tables:

1) add some tables of figures to make easily presentation to describe the study correctly.

We thank the reviewer for this suggestion. We agree that the original table, while comprehensive, placed a considerable burden on the reader to reconstruct the sequence and timing of study procedures. Following this recommendation, we have added some pictograms to convey the information more easily.

2) convert to diagram rather than in a tabe

We appreciate the reviewer's valuable comment. We have converted the table into a visual flowchart (now Figure 1) that presents the study procedures as three clearly delineated phases — the phone call prior to each in-person visit, the in-hospital laboratory assessment, and the post-visit. Since the procedure is identical across Visit 1 and Visit 2 (differing only in gestational trimester), this format also allowed us to present the workflow once rather than duplicating it across two columns, improving overall readability. We believe this revision makes the study design substantially easier for readers to follow and have updated the manuscript text accordingly to reference Figure 1.

Reviewer: 1
If you have selected 'Yes' above, please provide details of any competing interests.: Not applicable

Reviewer: 2
If you have selected 'Yes' above, please provide details of any competing interests.: Not applicable

---

VERSION 2 - REVIEW

**Reviewer**	**1**
**Name**	**Daneshvar, Mojtaba**

Affiliation **Tehran University of Medical Sciences, faculty of nutritional sciences and dietetics, Tehran University of Medical Sciences, Tehran, Iran**

Date **01-Aug-2026**

COI

I acknowledge that all requested revisions have been thoroughly and appropriately addressed in the revised manuscript.

Reviewer **2**

Name **Aji, Arif Sabta**

Affiliation **National Research and Innovation Agency Republic of Indonesia, Research Center for Biomedical**

Date **04-Aug-2026**

COI

The authors have addressed all the suggested comments from the reviewer.
